# The role of ADAR1 through and beyond its editing activity in cancer

**DOI:** 10.1186/s12964-023-01465-x

**Published:** 2024-01-17

**Authors:** Yue Jiao, Yuqin Xu, Chengbin Liu, Rui Miao, Chunyan Liu, Yilong Wang, Jiao Liu

**Affiliations:** School of Basic Medicine Sciences, Weifang Medical University, Weifang, 261053 China

**Keywords:** ADAR1, RNA editing, RNA editing-independent, Cancer, Cancer therapy

## Abstract

Adenosine-to-inosine (A-to-I) editing of RNA, catalyzed by adenosine deaminase acting on RNA (ADAR) enzymes, is a prevalent RNA modification in mammals. It has been shown that A-to-I editing plays a critical role in multiple diseases, such as cardiovascular disease, neurological disorder, and particularly cancer. ADARs are the family of enzymes, including ADAR1, ADAR2, and ADAR3, that catalyze the occurrence of A-to-I editing. Notably, A-to-I editing is mainly catalyzed by ADAR1. Given the significance of A-to-I editing in disease development, it is important to unravel the complex roles of ADAR1 in cancer for the development of novel therapeutic interventions.

In this review, we briefly describe the progress of research on A-to-I editing and ADARs in cancer, mainly focusing on the role of ADAR1 in cancer from both editing-dependent and independent perspectives. In addition, we also summarized the factors affecting the expression and editing activity of ADAR1 in cancer.

## Background

 Adenosine-to-inosine (A-to-I) RNA editing is a biological process that converts adenosine to inosine in double-stranded RNA (dsRNA) molecules. Inosine is recognized by the cell as guanosine and paired with cytosine. Therefore, the base conversion may affect the amino acid sequence of the protein. ADARs, a family of enzymes, act on double-stranded RNA (dsRNA) to catalyze this conversion. Among them, ADAR1 has been extensively studied in cancer, while ADAR2 and ADAR3 have received less attention, and ADAR3 has a total lack of activity [1]. ADAR1 is widely expressed throughout mammalian tissues and has been shown to modify substrates within both nuclear and cytoplasmic compartments [2]. Exploring the biological mechanisms of ADAR1 in cancer may provide important implications for clinical diagnosis and treatment.

In this review, we first provide information on the characters of ADAR1, and then we describe the roles that ADAR1 can promote or suppress cancer in RNA editing-dependent and independent manners. Finally, we summarize the modulation of the level and editing activity of ADAR1 by multiple factors.

## A-to-I RNA editing

Deamination of adenosine-to-inosine (A-to-I) is a widespread post-transcriptional modification of RNA and is catalyzed by Adenosine deaminase acting on RNA (ADAR) enzymes in dsRNA [3]. As early as 2004, it was reported that ADAR1 plays an important role in embryonic development by editing dsRNA to keep embryos alive and prevent stress-induced apoptosis [4]. A large number of A-to-I editing sites exist in the human genome which lays the foundation for transcriptome diversity and most editing occurs in the Alus sequence [5, 6]. With the development of high-throughput sequencing analysis, RNA editing has been seen in many diseases, especially in cancer. The RNA modification of A-to-I editing is significantly altered in many cancer types, most of which show increased editing and are associated with patient survival, and also affect drug sensitivity [7, 8].

Given the important roles of A-to-I editing in cancer, this RNA modification process is thought to be potentially involved in carcinogenesis as an epigenetic mechanism [9]. To more intuitively and accurately understand the activity of ADAR enzymes and the changes in A-to-I editing in organisms, scientists have developed and designed a series of tools to objectively reflect the level of A-to-I editing (Table 1). Many of them are bioinformatics algorithms, by which A-to-I editing sites can be identified based on transcriptome sequencing [10, 11]. To quantify ADAR activity, Roth et al. present the Alu editing index (AEI), a robust and simple-to-use computational tool, based on the fact that almost all human editing events occur in Alu repeats. They defined the AEI as the ratio of the number of A-G to the number of A-G and A-A at the RNA-DNA mismatch sites within human Alu repeats. Unlike previous approaches, the AEI offers a reliable, unbiased estimate of the editing level that averages over millions of sites, enables direct comparison of several samples, and is simple to use for any kind of organism. It can also enable us to investigate the factors influencing the global editing activity [12]. To circumvent the errors in RNA-seq data and make the detection of editing sites more accurate, researchers exploited EndoVIPER-seq to improve such problems [13]. Recently, Zhu et al. developed a network interactive server to facilitate users to study the relationship between A-to-I editing sites of interest and cancer [14]. Alternatively, a sensitive ADAR editing reporter can be stably introduced into the cell to determine the editing activity according to the fluorescent signal quantitatively [15].

**Table 1:** A-to-I RNA editing detection tools

Tool	URL	Description	Refs
AEI	https://github.com/a2iEditing/RNAEditingIndexer	A robust and simple-to-use computational tool can be used to quantify global ADAR1 editing activity.	[12]
REIA	http://bioinfo-sysu.com/reia	An interactive web server can be used to explore the relationship between A-to-I RNA editing sites and cancer.	[14]
SPRINT	http://sprint.tianlab.cn	An SNP-free method to recognize RNA editing sites (RESs).	[16]
RED-ML	https://github.com/BGIRED/RED-ML	Machine learning-based RNA editing detection.	[17]
FLAIR2	https://github.com/BrooksLabUCSC/flair	A tool can detect chaplotype-specific variant and transcript-specific RNA editing	[18]
RES-Scanner	https://github.com/ZhangLabSZ/RES-Scanner	A software package can be used to detect annotate RNA-editing sites.	[19]
RADAR	http://RNAedit.com	An A-to-I RNA editing database.	[20]
JACUSA	https://github.com/dieterichlab/JACUSA	A one-stop solution to detect single nucleotide variant sites and A-to-I RNA editing sites.	[21]
REDIportal	at http://srv00.recas.ba.infn.it/atlas/	The most extensive and complete database on human RNA editing.	[22]
MiREDiBase	https://ncrnaome.osumc.edu/miredibase	An editing event catalog in miRNAs.	[23]
FLARE	https://github.com/YeoLab/FLARE	A fast and flexible workflow to identify RNA editing sites.	[24]
MultiEditR	z. umn.edu/multieditr	The first tool to identify and measure RNA editing using Sanger sequencing.	[25]
RDDpred	http://biohealth.snu.ac.kr/software/	A software package to predict putatively real RNA-editing events by using RNA-seq data.	[26]
RNAEditor	http://rnaeditor.uni-frankfurt.de	A bioinformatics tool to identify RNA editing events.	[27]
iRNA-3typeA	http://lin-group.cn/server/iRNA-3typeA/	A web server can identify three types of RNA modification at adenosine sites.	[28]
ATTIC	http://web.unimelb-bioinfortools.cloud.edu.au/ATTIC/	An approach to predict RNA editing sites in different species.	[29]
DeepEdit	https://github.com/weir12/DeepEdit	A neural network model can recognizes A-to-I editing events and resolves its phasing on transcripts.	[30]

## ADARs isoforms and domain structures

**Fig. 1:**
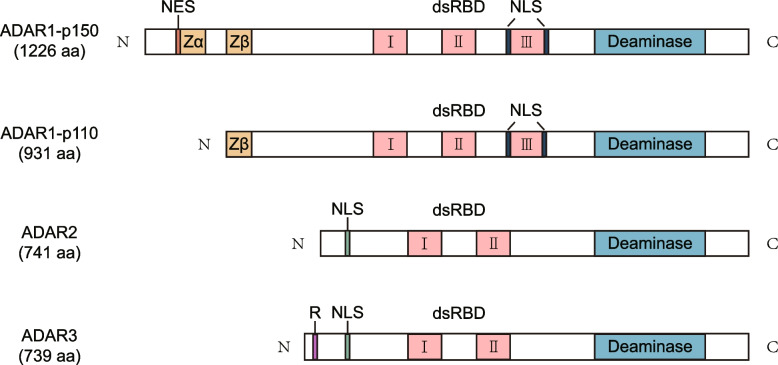
The domain structures of ADARs. All ADARs contain catalytically active deaminase domains and nuclear localization signal (NLS). There are two isoforms of ADAR1, p150 and p110, both of them contain a zβ domain, three dsRNA binding domains. ADAR1 p150 also contains a zα domain and a nuclear export signal (NES) at the N-terminal sequence. Compared to ADAR1, ADAR2 and ADAR3 have one fewer dsRNA binding domain. ADAR3 has an R-domain, which enables it to bind not only double-stranded RNA but also single-stranded RNA.

The three members of the ADAR family, ADAR1, ADAR2, and ADAR3, all contain highly evolutionarily conserved catalytic deaminase domain (DM) and three or two double-stranded RNA-binding domains (dsRBDs). ADAR1 is more specific because it has a part of the structure that is different from ADAR2 and ADAR3 (Fig. 1). As early as 1996, it was reported that ADAR1 contains two major isoforms, the interferon-inducible ADAR1 p150, and the constitutively expressed ADAR1 p110. ADAR1 p150 is localized both in the cytoplasm and nucleus, whereas ADAR1 p110 is mainly in the nucleus [31]. In the three dsRBD (RI, RII, and RIII) domains of ADAR1, scientists mutated each dsRBD separately to explore their effects on the binding activity of dsRNA and adenosine deaminase activity. The results showed that the binding activity of RNA would be greatly reduced after the mutation of RIII, so it is the most important of the three dsRBDs [32]. Additionally, RIII as an RNA-sensitive nucleocytoplasmic transport signal in ADAR1 can mediate nuclear import [33]. Nuclear localization signal (NLS) that overlaps the RIII domain, mediates nuclear import by interacting with the import receptor transportin 1 (Trn1). And the RIII domain can serve as a bridge between the two sides of the NLS structure, facilitating the localization of the NLS and the interaction with Trn1 [34, 35]. In addition, ADAR1 contains the Z-DNA/RNA binding domain (ZBD), also known as Zalpha (Zα) or Zbeta (Zβ), which is a 78-amino acid protein-fold structure that binds exclusively to Z-DNA and Z-RNA but not to B-DNA. ZBDs may play an important role in the innate antiviral immune response. Both ADAR1 isoforms contain a Zβ domain, but besides the Zβ domain, ADAR1 p150 contains a Zα domain [36–38]. There is a nuclear export signal (NES) in the Zα domain, which regulates ADAR1 p150 penetration out of the nucleus [39]. This also clarifies why the majority of ADAR1 p110, ADAR2, and ADAR3 are found in the nucleus rather than the cytoplasm.

In contrast to ADAR1, ADAR2 and ADAR3 have only two dsRBD structural domains and their NLS domains are localized to the N terminus. In addition, ADAR3 contains a unique R domain, which enables it to bind not only double-stranded RNA but also single-stranded RNA [1].

## RNA editing-dependent ADAR1 functions in tumors

### RNA editing in coding regions

Recently, the sequencing technologies advances rapidly, which enable the detection of millions of A-to-I editing sites in human [11, 20, 22]. At these sites, base changes may alter amino acid sequences during translation, largely affecting the translation of transcripts or even altering the localization and function of encoded proteins (Table 2).

**Table 2:** ADAR1-mediated A-to-I editing in coding RNAs

Genes	Cancer	Molecular Mechanisms	Refs	Substrate type
Promoting cancer				
AZIN1	Esophageal squamous cell carcinoma	Hyper-editing of AZIN1	[40]	Coding sequence
	Colorectal cancer	Type 1 interferon triggers the expression of ADAR1 to induce AZIN1 over-editing Over-edited AZIN1 promotes increased expression of ODC, OCT4, SOX2 and CD44v6 By accumulating ODC	[41–43]	Coding sequence
	Hepatocellular carcinoma	Over-edited AZIN1 undergoes cytoplasmic-to-nuclear translocation and inhibits the degradation of ODC and CCND1	[44, 45]	Coding sequence
	Endometrial cancer	Inhibition of ADAR1 activates the dsRNA sensing signaling pathway and increases the expression of the pro-apoptotic factors MDA5, RIG-I, and PKR	[46]	Coding sequence
	Prostate cancer	Over-edited AZIN1 causes its nuclear translocation by binding to the actin/myosin 9 complex	[47]	Coding sequence
	Gastric cancer	Hyper-editing of AZIN1	[48]	Coding sequence
	Non-small cell lung cancer	Over-edited AZIN1 undergoes cytoplasmic-to-nuclear translocation and inhibits the degradation of ODC and CCND1	[49]	Coding sequence
CDK13	Thyroid cancer	CDK13 editing enriches its localization at the nucleolus and induces changes in splicing patterns	[50]	Coding sequence
	Hepatocellular carcinoma	ADAR1 mediates CDK13 editing	[51]	Coding sequence
BLCAP	Hepatocellular carcinoma	Activation of AKT/mTOR signaling pathway	[52]	Coding sequence
	Cervical cancer	BLCAP editing loses the ability to inhibit Bcl2, Mcl-1, Survivin, and STAT3 activation	[53]	Coding sequence
FLNB	Breast cancer	FLNB editing enriches its localization at the cytoplasmic	[54]	Coding sequence
	Hepatocellular carcinoma	Hyper-editing of FLNB	[45]	Coding sequence
	Esophageal squamous cell carcinoma		[40]	Coding sequence
KPC1	Intrahepatic cholangiocarcinoma	Over-edited KPC1 impairs the affinity of KPC1 for NF-κB1 p105, reduces p105 to p50 ubiquitination and proteasome processing, and enhances oncogenic NF-κB signaling activity	[55]	Coding sequence
NEIL1	Multiple myeloma	NEIL1 editing enhances double-stranded DNA damage repair responses	[56]	Coding sequence
GLI1	Hepatocellular carcinoma	Enhancement of cancer cell differentiation, upregulation of stemness-related proteins, promotion of epithelial mesenchymal-diffusion transition (EMT), GLI1 nuclear translocation	[57]	Coding sequence
FAK	Lung adenocarcinoma	ADAR1 binding and editing FAK to increase the stability of FAK mRNA and upregulate FAK expression	[58]	Intron
MOK	Melanoma	The mechanism is not clear	[59]	Alu
DZIP3				
ZBTB11				
Suppressing cancer				
Gabra3	Breast cancer	Reduced cell surface expression of Gabra3 and inhibited AKT activation	[60]	Coding sequence
GLI1	Multiple myeloma	Regulating the output of the hedgehog signal	[61]	Coding sequence

In cancer, antizyme inhibitor 1 (AZIN1) is one of the most studied proteins for A-to-I editing in the coding region. Initially, studies on the role played by over-edited AZIN1 were performed in liver cancer. They found that A-to-I editing occurs at position 367, which results in a serine-to-glycine substitution. The edited form of AZIN1 gains higher protein stability, and stronger affinity to antizyme, and undergoes cytoplasmic-to-nuclear translocation, which promotes cell proliferation [44]. Then, Ghalali et al. reported that the nuclear translocation of edited AZIN1 is dependent on binding to the actin/myosin9 complex, which can enhance cellular aggressiveness and is associated with worse outcomes of prostate cancer [47]. Recently, studies on RNA editing of AZIN1 have gradually increased and expanded to other cancer types, such as colorectal cancer, esophageal cancer, endometrial cancer, gastric cancer, lung cancer, etc. (Table 2).

By analyzing RNA and DNA data, Hu et al. found that Bladder cancer-associated protein (BLCAP) was also over-edited in liver cancer tissues, and over-edited BLCAP showed a cell proliferation promoting phenotype both in vivo and in vitro, which may be achieved by activating the AKT/mTOR signaling pathway [52]. Notably, over-edited GABA_A_ receptor alpha3 (Gabra3) can inhibit AKT activation, thereby suppressing breast cancer progression [60]. In addition, similar findings regarding the phenomenon that over-edited BLCAP promotes cancer progression were obtained in another study on cervical cancer. It was shown that wild-type BLCAP could inhibit the phosphorylation of signal transducer and activator of transcription 3 (STAT3) by directly interacting with it, whereas over-edited BLCAP lost its ability to inhibit STAT3 activation, which in turn promoted cancer progression [53].

There are still some unmentioned studies on the action of ADAR1-mediated RNA editing in the coding region. Here, we compile recent studies on the impact of over-editing proteins on cancer progression (Table 2).

### RNA editing in non-coding regions (3’UTR, Alus, miRNA, etc.)

ADAR1-mediated A-to-I editing is also important for the regulation of non-coding RNAs. Although editing of non-coding RNAs cannot affect protein translation by directly changing codons, it can indirectly regulate protein expression through other pathways.

#### In 3’ untranslated region

It is well established that the transmission of genetic information from mRNA to protein comes from the correct translation of the coding sequence on mRNA, and the untranslated regions (UTRs) at the 5’ and 3’ ends of mRNA have a complex role in regulating its stable translation. Currently, a large number of studies have shown the existence of multiple RNA modification types within the 3’ UTR, including A-to-I editing [62]. A double-stranded structure formed by the inverted-repeat Alus (IR-Alus) are often found in 3’UTR, which is more likely to recruit ADAR1 and undergo an A-to-I transition. This editing can regulate the localization, translation, and stability of mRNA [63]. In addition, A-to-I editing occurring on IR-Alus can also neutralize its immunogenicity, thereby functioning as a specific negative regulator of the MDA5-MAVS pathway, which prevents the production of type I interferon (IFN-I) [64–66].

By analyzing the transcriptomic data from the tumor and its paired “normal” tissues, researchers found that the number of editing sites and editing levels of some known sites in 3’UTR are both significantly increased [67]. We have compiled current studies of A-to-I editing on 3’UTR (Table 3).

**Table 3:** ADAR1-mediated A-to-I editing in non-coding RNAs

Genes	Cancer	Molecular Mechanisms	Refs	Substrate type
Promoting cancer				
DHFR	Breast cancer	miR-25-3p and miR-125a-3p bind to the unedited 3’-UTR of DHFR, but not to the edited DHFR	[68]	3’UTR
METTL3	Breast cancer	ADAR1 editing METTL3 mRNA alters its miR-532-5P binding site and upregulates METTL3, which in turn promotes recognition of ARHGAP5 by methylated YTHDF1	[69]	3’UTR
MDM2	HeLa ,HepG2 ,HEK293	ADAR1 competes with STAU1 to occupy the Alu element in the 3’UTR of XIAP and MDM2 to promote nuclear retention	[70]	3’UTR
XIAP				
PHACTR4	Gastric cancer	PHACTR4 Loss of the seed sequence required for miRNA-196a-3p binding	[71]	3’UTR
ATM	Breast cancer	Increased editing of 3′ UTRs of ATM, GINS4, and POLH transcripts. Editing GINS4 transcripts primarily regulates their stability, while editing ATM and POLH primarily regulates their expression	[72]	3’UTR
GINS4				
POLH				
ARHGAP26	SW480, Raji, DU145, U2OS, A549, MCF-7, MCF-10 A, HepG2, and Chang Liver cells	Disruption of miR-30b-3p and miR-573 binding sequences within the 3′ UTR of ARHGAP26	[73]	3’UTR
GM2A	Glioblastoma	Upregulating GM2A expression and promoting gemness	[74]	3’UTR
miR-200b	Multiple types of cancers	Impaired ability to inhibit ZEB1/ZEB2	[75]	MiRNA
miR-3144-3P	Liver cancer	Inducing the expression of MSI2 and suppressing the expression of SLC38A4	[76]	MiRNA
CircCHEK2	Multiple types of cancers	Editing of CircCHEK2 flanking introns can alter the stability of the RCM structure as well as regulate circRNA production by affecting the binding of RBP to flanking intron sequences.	[77]	CircRNA
hsa_circ_0004872	Gastric cancer	The mechanism is not clear	[78]	CircRNA
LINC00624	Breast cancer	Inhibiting type I interferon	[79]	LncRNA
Telomeric RNA:DNA hybrids	Hela	Correction of A-C mismatched bases in telomeric repetitive RNA: DNA hybrids facilitate their resolution by RNaseH2	[80]	R-loop
Suppressing cancer				
DFFA	Breast cancer	miR-140-3p directly targets the apoptosis-inducing gene DFFA in MCF-7	[81]	3’UTR
ARPIN	Breast cancer	Ectopic expression of miR-1285-3p, miR-1285-5p, and miR-619-5p interacts with sequences near the editing site to suppress its expression	[82]	3’UTR
miR-378a-3p	Melanoma	Preferential binding of the 3’-UTR of the Parva oncogene	[83]	MiRNA
miR-455-5p	Melanoma	Loss of affinity to CPEB1	[84]	MiRNA
miR-376a*	Glioblastoma	The target gene changed from RAP2A to AMFR	[85]	MiRNA
PCA3	Prostate Cancer	Editing PRUNE2/PCA3 dsRNA Regulates PRUNE2 Levels	[86]	LncRNA

*is used to indicate that this miRNA is derived from the opposite arm of the precursor

#### In Alus

Among transposable elements (TES), Alus is the most abundant repetitive element, accounting for about 10% of the human genome. The dsRNA structure of Alus facilitates the recruitment of ADAR1 for A-to-I editing [5, 87]. It was shown that A-to-I editing occurs predominantly in Alus sequence (> 90%) and is mainly catalyzed by ADAR1. It had been demonstrated that the endogenous “self” Alus sequence can be converted to “nonself” sequence by forming stem-loop structures, the latter can be sensed by melanoma differentiation-associated protein 5 (MDA5), which lead to the production of IFN-I. ADAR1-mediated A-to-I editing can neutralize the immunogenicity of Alus by disrupting the formation of stem-loop, converting the aberrantly expressed “nonself” Alus sequence into a “self” sequence, which avoids its stimulation of MDA5 and inhibits the expression of IFN-I regulated by the MDA5/MAVS/IRF7 signaling pathway [66, 88, 89]. Meanwhile, researchers found that IFN-I activated by the immunogenicity of Alus plays a key role in tumor immune surveillance, which could make the tumor more vulnerable to inhibition of ADAR1. This suggests that ADAR1 can serve as a new target in tumor therapy [90–92].

#### In MicroRNA

MicroRNAs (miRNAs) are known to be non-coding RNAs whose main function is to target the 3’UTR of mRNAs to degrade them and/or inhibit their translation [93]. The binding of miRNA to 3’UTR of mRNAs can be intervened by A-to-I RNA editing, which occurs on 3’UTR of mRNAs or miRNA (Table 3). For example, miR-25-3p and miR-125a-3p can bind to unedited Dihydrofolate reductase (DHFR) but not edited-DHFR, leading to upregulation of DHFR, which can increase tumor resistance to methotrexate and promote tumor proliferation [68]. Besides, miRNAs themselves undergo A-to-I editing and alter their binding preference. For example, in metastatic melanoma, edited but not unedited miR-378a-3p was found to target the 3’UTR of the oncogene PARVA to suppress its expression [83]. ADAR1, an enzyme that specifically recognizes dsRNA, can regulate miRNA maturation at various critical nodes of miRNA processing maturation. It can target the dsRNA structure of certain pri-miRNAs, causing them to undergo editing, thereby inhibiting the processing of pri-miRNA by RNase III DROSHA and reducing miRNA levels [94]. In addition, ADAR1 can also regulate miRNA biological processes by directly interacting with DROSHA and/or DGCR8 [95].

#### In CircRNA and LncRNA

In recent years, the study of ADAR1 in cancer has gradually expanded to circular RNA (circRNA) and Long noncoding RNA (lncRNA). CircRNA is considered a unique epigenetic regulatory molecule involved in the cancer process. After the booming development of high-throughput sequencing technology and bioinformatics, the biological significance of circRNA has gradually been elucidated [78]. The most important function of circRNA is to act as miRNA sponges, which can enhance gene expression levels by derepressing miRNAs on their target genes [96]. Recently, studies have reported that ADAR1 can reduce circRNA biogenesis by interacting with RNA helicase DHX9 and disrupting the formation of double-stranded RNA or by altering the secondary structure of the precursor of circRNA [97, 98]. Additionally, there is also research that hsa_circ_0004872 can inhibit ADAR1 expression through hsa_circ_0004872/miR-224/Smad4/ADAR1 negative feedback loop and exert oncogenic effects in gastric cancer (GC) [78].

Long noncoding RNAs (LncRNAs) are non-coding RNAs with more than 200 nucleotides that are involved in the regulation of a variety of biological processes in cancer [99, 100]. Some lncRNA also undergo ADAR1-mediated A-to-I editing in cancer. For instance, in prostate cancer, Prostate cancer antigen 3 (PCA3), a lncRNA, can form a double-stranded RNA with PRUNE2 (a human homolog of the Drosophila prune gene) to attract ADAR1 binding and exert an editing effect to regulate PRUNE2’s level [86].

## RNA editing-independent ADAR1 functions in tumors

### Acting in a protein-protein interaction manner

Although most of the studies on ADAR1 have been conducted on its editing function, the editing-independent role of ADAR1 has been successively confirmed by studies [101]. Currently, most of the studies on ADAR1’s editing-independent role in cancer are related to the processing of miRNA.

**Fig. 2:**
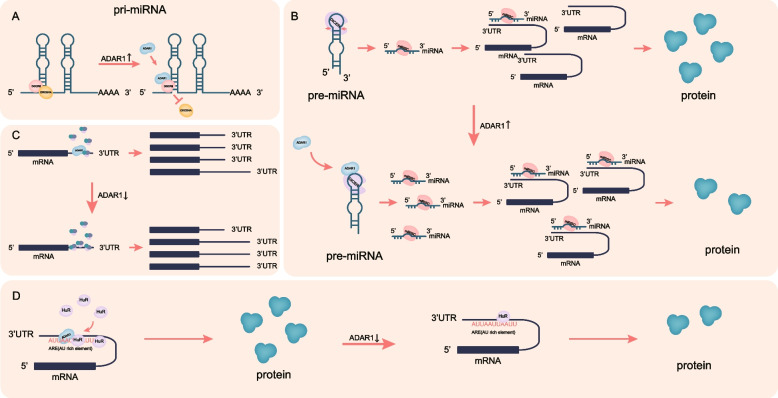
Editing-independent functions of ADAR1. **A** ADAR1 forms a complex with DGCR8 and interferes with DGCR8-DROSHA complex formation. **B** ADAR1 binds to DICER, accelerating the cleavage of pre-miRNAs to promote miRNA maturation, the later targets mRNAs, and downregulates protein levels. **C** ADAR1 competes with 3’UTR-binding factors for binding to the 3’UTR. When ADAR1 is downregulated, these proteins may have greater access to the 3'UTR, which could result in 3'UTR lengthening. **D** ADAR1 facilitates the binding of HUR to its target transcripts and increases transcript stability, which in turn enhances the translation efficiency of its target mRNAs

miRNA maturation requires a series of processes. In the nucleus, primary miRNA (pri-miRNA) is transcribed by RNA polymerase II and is processed by the DROSHA-DGCR8 complex, through which precursor miRNA (Pre-miRNA) with about 70 nucleotides is generated. Pre-miRNA is then exported to the cytoplasm where it can be further processed by the DICER enzyme into a double-stranded miRNA. Subsequently, one miRNA strand of the double-strand is degraded and the other is the mature miRNA. By binding to 3’UTR of the target mRNA, the mature miRNA can mediate its degradation [102]. During this process, the function of the three critical proteins, DROSHA, DGCR8, and DICER, are influenced by ADAR1 in an editing-independent manner. For example, ADAR1 can form a complex with DGCR8 interfering with the formation of the DGCR8-DROSHA complex [103] (Fig. 2A). ADAR1 can also promote the degradation of DROSHA by enhancing its ubiquitination [104]. In addition, ADAR1 can form a heterodimer with DICER, which accelerates the cleavage of pre-miRNA and promotes the loading of miRNA-induced silencing complex (miRISC) onto 3’UTR of the target mRNA, thereby regulating the expression of target genes [105–107] (Fig. 2B). Notably, ADAR1 can also regulate the expression level of DICER by regulating the level of let-7 [103].

### Acting as RNA binding protein

ADAR1, as an RNA-binding protein, acts directly on RNA to perform some editing-independent functions. Besides the aforementioned interactions of ADAR1 with some key proteins in miRNA processing, ADAR1 can also directly bind to pri-miRNA, which regulates the maturation of miRNA [108]. Furthermore, ADAR1 competes with some 3’UTR processing factors for “occupancy” of the 3’UTR to be involved in the processing [95] (Fig. 2C).

ADAR1 and other RNA-binding proteins can jointly regulate gene expression by binding to the same RNA substrates. For instance, the interaction of ADAR1 with nuclear factor 90 (NF90) family proteins rely on the bridge of dsRNA and mediates gene expression [109]. Human antigen R (HuR) is another RNA binding protein, it can specifically bind the ARE element (AU-rich element) of target mRNA and upregulates its expression by increasing the stability and translation efficiency of target mRNA [110]. During this process, ADAR1 can exert its function by cooperating with HuR [111] (Fig. 2D). Similarly, it has also been shown that ADAR1 can bind to cyclin-dependent kinases 2 (CDK2) mRNA to exert a pro-oncogenic effect, even though the deeper mechanisms behind this are not clear. However, it is certain that none of these processes is dependent on the editing activity of ADAR1 [108, 112].

These findings highlight the versatility of ADAR1 and suggest that it may have multiple roles beyond its well-characterized A-to-I RNA editing function. In summary, ADAR1 possesses both editing-dependent and independent functions in tumors, which exert diverse effects on various biological processes. Further studies are required to elucidate the precise molecular mechanisms underlying these functions and their implications for disease pathogenesis and therapeutic development.

## Influencing factors of ADAR1 expression and activity

### Regulation of ADAR1 through post-transcriptional or post-translational modification

As the coding gene, ADAR1 modifications that occur at the mRNA level or protein level can have an impact on its expression, localization, function, etc. In a recent study on glioma, researchers found that RNA methyltransferase-like 3 (METTL3) can target the m6A site near the termination codon of the ADAR1 transcript to regulate ADAR1 protein expression thereby affecting the proliferation of glioma cells [112].

**Fig. 3:**
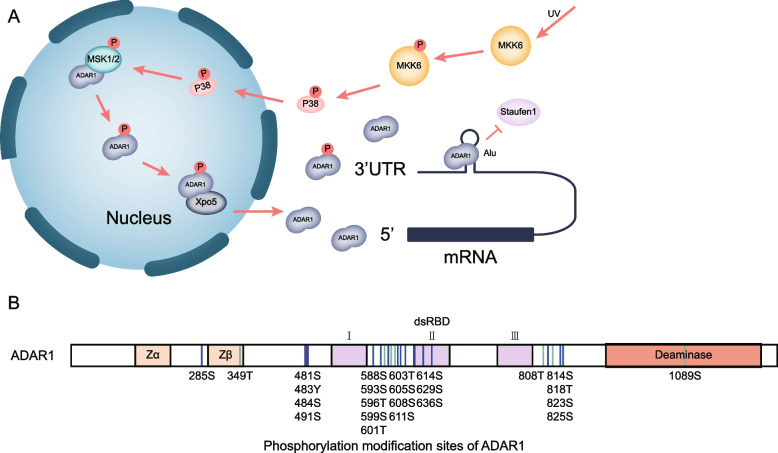
The phosphorylation of ADAR1. **A** Phosphorylation of ADAR1 by MKK6–p38–MSK1/2 mitogen-activated protein (MAP) kinase promotes its binding to Exportin-5 (Xpo5), which exits the nucleus and competes with Staufen1 for the 3'-UTR of antiapoptotic genes. **B** Phosphorylation modification sites of ADAR1 (from UniProt: https://www.uniprot.org/uniprotkb/P55265/entry, positions source: UniProt and PRIDE S: phosphoserine, T: phosphothreonine, Y: phosphotyrosine)

The impact of post-translational modifications (PTMs) on the function of the protein itself and the interaction with other molecules cannot be ignored. Common types of modifications include phosphorylation, ubiquitination, glycosylation, and palmitoylation [113]. Among them, phosphorylation is one of the most studied modifications in PTMs, which can phosphorylate some proteins to regulate their localization, conformation, and activity [114]. It has been shown that MKK6–p38–MSK1/2 mitogen-activated protein (MAP) kinases phosphorylate two threonine sites and three serine sites (T808, T811, S814, S823, and S825) of ADAR1, and tethers it to the nuclear export protein Exportin-5 to facilitate its nuclear exportation. Then, ADAR1-p110 can compete with Staufen1 for the 3’UTR of some antiapoptotic transcripts to participate in the stress response and protect cells from apoptosis under stress conditions. Notably, the aforementioned function is independent of its RNA editing activity [115] (Fig. 3A). Akt (Protein Kinase B) activity has been reported to be associated with several cancer processes [116]. Recently, Alberto Bavelloni et al. showed that ADAR1 p110 can act as a substrate for Akt kinase, and phosphorylation at T738 of ADAR1 p110 can significantly reduce its editing activity, which in turn influences the progression of editing-related diseases [117]. According to the information from the Uniprot website (https://www.uniprot.org/uniprotkb/P55265/entry), we note that the phosphorylation sites of ADAR1 are not evenly distributed. Among them, most of the sites tend to be distributed on unknown functional domains and are densely distributed in the region where the RI and RII structural domains join. This suggests that it may be a phosphorylation-dependent functional regulatory region that influences protein structure, activity, and function. So far, there are only four phosphorylation sites distributed in the known functional domains of ADAR1. Three of these phosphorylation sites are located within the second dsRNA binding domain (RII), and one is in the deaminase domain. Therefore, it suggests that modification of these sites affects the binding of ADAR1 to some double-stranded RNAs and may also affect their activity and function (Fig. 3B). In addition, modification of ubiquitination has also been reported. The E3 ubiquitin ligase SMURF2 binds directly to ADAR1 p110, which ubiquitinates the lysine at position 744, increases its stability, and promotes A-to-I editing activity [118]. Sumoylation is a post-translational modification of proteins similar to ubiquitination. SUMO-1, a ubiquitin-like protein modification molecule, can modify the lysine residue at position 418 of ADAR1 and be found to reduce the editing activity of ADAR1 [119].

### Impact of protein-protein interaction on the catalytic activity of ADAR1

Both homodimerization and heterodimerization can be formed between ADAR1 and ADAR2, which is extremely important for the activity of the ADAR family [120]. Other proteins in the cell can also act on ADARs, thereby affecting their activity. For example, death-associated protein 3 (DAP3) is considered as an editing repressor, it can inhibit A-to-I editing in cancer cells by interfering with the homodimerization of ADAR1 and inhibiting the binding of ADAR2 to dsRNA [121]. In addition, cytoplasmic polyadenylation element-binding protein 3 (CPEB3) inhibits ADAR1-mediated RNA editing by localizing ADAR1 mRNA to the processing body (P-body), inhibiting the translation of ADAR1, thereby suppressing gastric cancer progression [122]. It has been shown that in the nucleus, DDX6 interacts with ADAR1 to regulate A-to-I editing, and the C-terminal domain or nuclear entry features of DDX6 were found to repress ADAR1 activity [123].

It is noteworthy that recent research has shown that Long non-coding RNAs (LINC00624) can also affect the stability of ADAR1 by binding to it [79].

### Impact of ADAR1 by other factors

It has been reported that a high level of editing can be triggered by the presence of an Edit Inducing Element (EIE) in the vicinity of the editing sites, which can form long double-stranded structures that are easily hyper-editing, recruiting ADAR1, thereby increasing editing efficiency [124].

While the secondary structure (dsRNA) is more important for ADAR1 to bind, the base pair of the duplex RNA adjacent to editing sites also influence the editing efficiency. For example, study had shown that a non-Watson–Crick pairing of guanosine (G: A or G: G) 5′ nearest neighbor of editing sites in duplex RNA can facilitate ADAR editing [125, 126].

It has also been reported that intracellular acidification enhances RNA editing by increasing ADAR1 base flipping and deamination rates [127].

## Conclusions and prospects

A-to-I editing is one of the most studied types of RNA modifications to date, especially today with the rapid development of high-throughput sequencing technologies and bioinformatics. A series of tools have been developed to detect editing sites and to predict the relevance of editing to cancer [13, 14].

In this review, we briefly summarize studies related to the involvement of ADAR1 in cancer progression from both editing-dependent and editing-independent perspectives. We also describe a number of factors that affect ADAR1’s expression and activity, including ADAR1’s own modifications (post-transcriptional and post-translational modifications), interactions of other proteins with ADAR1, the primary sequence and secondary structure of the substrates (RNAs) of ADAR1, and the effect of the microenvironment on its function.

Currently, the role of ADAR1 in tumor immunotherapy is receiving attention. For example, the editing of Alus by ADAR1 reduces its immunogenicity, which in turn reduces the body’s tumor immune surveillance and tumor immunotherapy effects [64]. The Warburg effect is widely present in cancerous tissues. Currently, a large number of abnormalities and mutations in metabolic enzymes have been identified in many cancer types, most of which may directly or indirectly lead to an increase of lactate that acidifies the extracellular environment of the tumor [128]. In addition, it has been shown that the acidic tumor microenvironment can increase A-to-I editing levels [127, 129]. Thus, it suggests that lactic acid may be involved in cancer development as a potent editing inducer to reduce the immunogenicity of Alus thereby suppressing tumor immune surveillance. This provides a new clue for immunotherapy by targeting ADAR1, and further studies on the factors affecting ADAR1 activity are expected to provide new strategies for tumor immunotherapy.

Additionally, aberrant A-to-I editing is commonly found in cancers, where over-editing of some sites is accompanied by under-editing of others. For example, high editing of the FLNB and low editing of the COPA are present in hepatocellular carcinoma (HCC). Both of them can drive the development of HCC [45]. Currently, several site-directed RNA editing (SDRE) systems utilizing endogenous or exogenous ADARs have been developed and have attracted the attention of scientists due to their biological tolerance and greater biosafety [130–132]. It has been shown that SDRE can restore the activity of TP53 by correcting its pathogenic mutations [133]. Thus, the SDRE system may be a promising candidate in tumor therapy. However, the strategies of targeted RNA A-to-I editing by using endogenous or exogenous ADARs are both limited. For example, endogenous ADARs expression levels are insufficient, and delivered exogenous ADARs reduce their substrate activity [134]. Therefore, detailed understanding the factors that influence ADARs editing activity is essential to enhance their activity. In addition, in tumors, there is usually a large of number aberrant editing sites, many of which have been demonstrated to be involved in the progression of the disease. Therefore, correcting single-site editing abnormalities is often not sufficient for therapeutic needs, and the development of tools for multisite editing has still to be explored to provide new strategies for the treatment of tumors.

## Data Availability

Not applicable.

## Declarations

The authors declare that their participation in writing this review as well as its publication is completely voluntary without afecting their actual research work.
